# First week of life respiratory management and pulmonary ventilation/perfusion matching in infants with bronchopulmonary dysplasia: a retrospective observational study

**DOI:** 10.1038/s41372-022-01569-1

**Published:** 2022-12-02

**Authors:** Malin Kjellberg, Alejandro Sanchez-Crespo, Baldvin Jonsson

**Affiliations:** 1 Department of Woman and Child Health, Department of Neonatology, Karolinska Institute, Stockholm, Sweden; 2 Department of Oncology-Pathology, Karolinska Institute, Stockholm, Sweden; 3 Department of Medical Radiation Physics and Nuclear Medicine, Karolinska University Hospital, Stockholm, Sweden

**Keywords:** Diagnosis, Predictive markers

## Abstract

**Objective:**

To investigate the association between early neonatal respiratory management in infants with bronchopulmonary dysplasia (BPD) and the degree of pulmonary ventilation perfusion-matching (V/Q) at term.

**Methods:**

30 preterm infants with a diagnosis of BPD who were initially treated with either controlled mechanical ventilation/continuous positive airway pressure (CMV/CPAP) (*n* = 14) or high-frequency oscillatory ventilation (HFOV) using a high lung-volume strategy (*n* = 16) were retrospectively included in this study. All infants underwent pulmonary V/Q single photon emission computed tomography at a median postmenstrual age of 37 weeks.

**Results:**

Infants treated with HFOV had significantly larger proportion of the lung with matched V/Q as compared to infants treated with CMV/CPAP, median (interquartile range) 60.4% (55.5–66.0%) and 45.8% (37.8–53.1%) respectively (*p* = 0.01).

**Conclusions:**

In infants who needed mechanical ventilation the first week of life and later developed BPD an association was observed between treatment with a HFOV and better pulmonary V/Q matching at near-term age.

## Introduction

Bronchopulmonary dysplasia (BPD) is one of the most common major morbidities among extremely preterm infants and remains a major challenge in the field of neonatology [1]. Treatment for BPD needs to be aimed at prevention and reduction of disease severity. The use of nasal continuous positive airway pressure (nCPAP) for spontaneously breathing very low birth weight infants has been shown to decrease the incidence of BPD [2]. However, a significant number of infants may fail nCAP and require mechanical ventilation [3]. Besides gestational age, the initiation of mechanical ventilation is the strongest risk factor for later BPD development and invasive ventilator management strategies and modalities vary between neonatal centers [4, 5]. Ventilator-induced lung injury is caused by volutrauma, atelectotrauma, and biochemical injury with a strong component of an ensuing inflammatory response [6, 7].

Single Photon Emission Computed Tomography (SPECT) is a non-invasive method enabling functional in vivo imaging of a pathophysiological process. The regional distribution of the pulmonary ventilation (V) and perfusion (Q) mapped with SPECT has been used in adults, children, and infants to quantify the proportion of lung functional loss due to lung regions of V/Q mismatching [8–11]. The aim of this study was to correlate respiratory support management during the first week of life with pulmonary V/Q matching at near-term age. Our hypothesis was that the degree of pulmonary V/Q abnormalities at near-term age is associated with the type of respiratory management during the first week of life in BPD patients.

## Patients and methods

### Study population

**Fig. 1:**
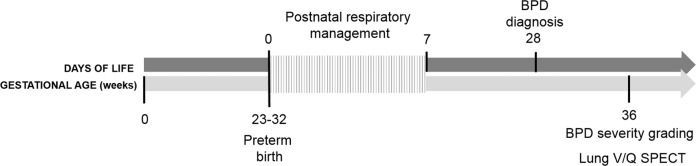
Flow diagram of the study design. The detailed timeline highlights the clinical course and SPECT examination in relation to the infants postmenstrual age.

Thirty preterm patients were retrospectively accrued from a prior study on BPD infants who underwent V/Q SPECT-scan examination at near-term age [11]. All patients were treated at the Karolinska University Hospital units from 2006 to 2010 and had a diagnosis of BPD based on the 2001 National Institutes of Health criteria [12]. The patients were then reclassified for severity of BPD according to the newest 2019 BPD definition [13]. The median age at SPECT examination was 37 weeks postmenstrual age. Figure 1 shows the timeline for this study. The regional ethical and radiation protection committees of the Karolinska University Hospital, Stockholm, Sweden approved the study. Informed parental consent was obtained for all patients included in the study. For these patients the following perinatal variables and variables related to the respiratory treatment in the postnatal period were recorded during the first week of life as well as for the entire stay in the neonatal intensive care unit (NICU); antenatal steroid treatment, preterm premature rupture of membranes (pPROM), delivery mode, gestational age, birth weight, weight Z-score, clinical risk index for babies (CRIB II score), small for gestational age (SGA), maximum and minimum partial pressure of carbon dioxide (PaCO_2_), partial pressure of oxygen (PaO_2_), maximum oxygen requirement (FiO_2_), daily maximum mean airway pressure (MAP), oxygenation Index (OI), age at intubation, days with mechanical support. Outcome data at discharge from ICU was collected for major morbidities (retinopathy of prematurity (ROP), intraventricular hemorrhage (IVH) grade 3 or more, necrotizing enterocolitis (NEC), late-onset sepsis until discharge and total time with respiratory support.

### Treatment groups

At the time this cohort was recruited, the local clinical guidelines for postnatal respiratory management included:

i. Start on nasal continuous positive air pressure (CPAP) with 5–8 cm H_2_O directly after delivery if breathing spontaneously.
ii. Intubation and 100–200 mg/kg surfactant (Curosurf) if not breathing.
iii. Impending CPAP failure was defined as increasing FiO_2_ > 0.4, X-ray with RDS or decreased lung volume, respiratory acidosis with pH < 7.2, frequent apnea or respiratory distress not responding to an increase in CPAP pressure.
iv. If breathing spontaneously with RDS and failing CPAP same as point ii with a clinical choice of either intubation/extubation (INSURE) or directly to MV.
v. If mechanical ventilation was needed the choice of mode was ultimately chosen by the attending physician. Recommended modes were high-frequency oscillatory ventilation (HFOV), or conventional mechanical ventilation (CMV) as synchronized intermittent positive pressure ventilation (SIPPV). Permissive hypercapnia was allowed if pH > 7.2.
vi. For HFOV a high lung volume strategy was used with an initial continuous distending pressure (CDP) of 10–12 cm H_2_O, then adjusted at 10–15 min intervals for optimal lung expansion based on oxygenation response and chest-X-ray. Carbon dioxide (CO_2_) elimination was managed by adjusting amplitude based on partial pressure of carbon dioxide (PaCO_2_) levels, either arterial or transcutaneous. Frequency was initially set at 10–15 Hz depending on birth weight.
vii. SIPPV was used for CMV. Tidal volumes of 5–7 ml/kg were recommended, but volume guarantee was not universally used. Peak inspiratory pressure (PIP), end-expiratory pressure (PEEP), and backup rate were adjusted to achieve satisfactory lung expansion and CO2 elimination.

**Fig. 2:**
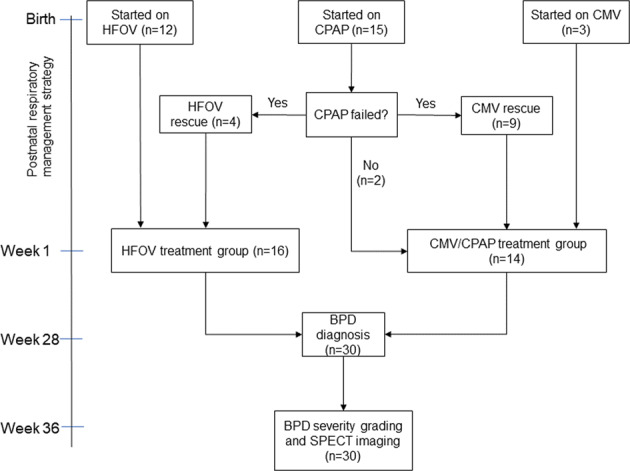
Flow diagram showing the original disposition of respiratory management for the 30 infants included in this cohort. At the top the initiation of respiratory support depends on whether the infant can breathe spontaneously or not. The final box shows, for these patients, the time for BPD grading and SPECT examination at near-term age.

In accordance with these guidelines, the 30 patients included in this cohort were divided in two treatment groups depending on the respiratory management used during the first week of life as described in Fig. 2. A total of 16 infants started on or were rescued after CPAP failure with HFOV using a high lung-volume strategy and 14 infants started on or were rescued with CMV or were only treated with CPAP.

### Outcome variables

The need for respiratory support and the degree of pulmonary V/Q matching at 36–37 weeks postmenstrual age were selected as the main outcome variables for the treatment groups.

### SPECT-scan and V/Q matching quantification

SPECT scans were performed at the Department of Nuclear Medicine, Karolinska University Hospital [11, 14]. A three-headed gamma camera (TRIAD XLT, Trionix Research laboratory, Twinsburg, OH, United States) equipped with low-energy, high-resolution plane parallel-hole collimators was used. With the patient immobilized in the camera bench with a vacuum bag, ~3 Mega-Becquerel (MBq) of Technetium 99 m labeled macro aggregate albumin (Tc99m-MAA; Mallinckrodt Medical, Petten, The Netherlands) were intravenously administered and directly followed by the first SPECT-scan. Thereafter, and with the infant lying in the same position, the ventilation SPECT-scan was started directly after administration of ~5 MBq of Technegas aerosol (Tetley Manufacturing Ltd. Sydney, Australia) by facemask during normal tidal breathing. Oxygen saturation and heart rate were continuously monitored with a pulse oximeter. The patients received ~1 milli-Sievert effective dose.

Prior to SPECT image reconstruction, the contribution from Tc99m-MAA in the ventilation SPECT-scan was removed by pixel-wise subtraction. All SPECT scans were reconstructed using an ordered subset expectation maximization (OSEM) algorithm. Three sets of reconstructed images were then generated, V, Q, and combined V + Q. The total functional lung volume was first obtained using a region-growing algorithm with a seed located at the mid-section of each of the lungs in the reconstructed V + Q images. This 3D-map was subsequently used to delineate the individual V and Q images. The final segmented V and Q images were normalized to their respective average and the V/Q ratios pixel-wise were calculated. In this work, we use the proportion (%) of the lung with matched V/Q ratios in the interval [0.6–1.4], where active gas exchange is occurring as the main outcome.

### Statistical analysis

The medians and interquartile ranges of the numerical variables were used as descriptive statistics. Due to the small size of this cohort, the Fisher exact test was used for statistical inference between treatment groups with regard to nominal variables and the two-tailed Mann-Whitney *U* test was used for statistical inference between treatment groups with regards to numerical variables. A two-way nonparametric Anova with interaction test was used to compare the pulmonary V/Q matching between the two respiratory treatment groups across all BPD subgroups. Perinatal factors describing the infant’s initial presentation that were significantly different between respiratory treatment groups, were included in a multivariate linear regression analysis to further evaluate their possible confounding effect on the relationship between the type of respiratory treatment and V/Q outcome.

## Results

**Fig. 3:**
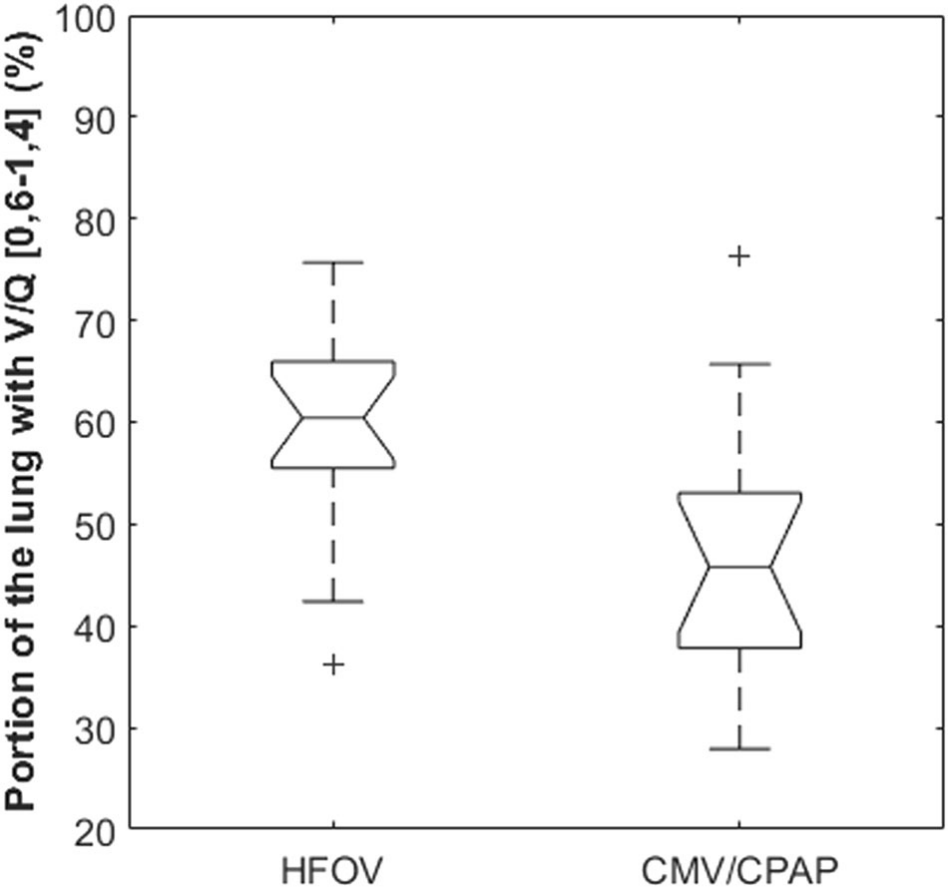
Primary outcome of pulmonary ventilation/perfusion (V/Q) matching at 36-37 weeks postmenstrual age. The boxplots show the proportion (%) of the lung with matched V/Q ratios in the interval [0.6,1,4] for BPD patients stratified according to respiratory treatment at first week of life with high-frequency oscillatory ventilation (HFOV) or controlled mechanical ventilation/continuous positive airway pressure (CMV/CPAP).

**Fig. 4:**
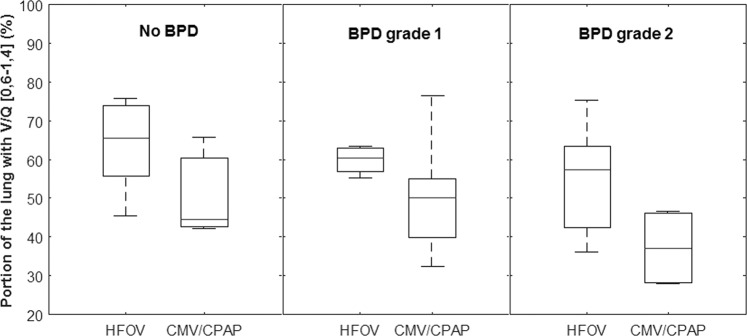
Primary outcome of pulmonary ventilation/perfusion (V/Q) matching at 36-37 weeks postmenstrual age. The boxplots show  the proportion (%) of the lung with matched V/Q ratios in the interval [0.6,1,4] for the patients stratified by both, the severity of BPD according to Jensen’s criteria [13] and treatment strategy at first week of life with HFOV or CMV/CPAP. Median and interquartile range V/Q matching for the HFOV and the CV/CPAP groups were respectively 65.5% (55.7–73.8%) and 44.5% (42.7–60.4%) for patients with no BPD, 60.4% (56.9–62.9%) and 50.2% (39.8–55.1%) for patients with BPD grade 1 and 57.3% (42.4–63.5%) and 37.1% (28.1–46.2%) for patients with BPD grade 2.

Table 1 shows that there was no significant difference between the treatment groups with regard to need for respiratory support at 36–37 weeks postmenstrual age (*p* = 0.43). Table 2 shows no significant statistical differences between the two respiratory treatment groups with regard to major neonatal morbidities or time with respiratory support. However, Table 1 and Fig. 3 reveal that at near-term age, patients in the HFOV treatment group had statistically significantly better pulmonary V/Q matching as compared to the CMV/CPAP group, median and interquartile range 60.4% (55.5–66.0%) and 45.8% (37.8–53.1%), respectively, *p* = 0.01. Further, Fig. 4 shows the distribution of pulmonary V/Q matching stratified according to respiratory treatment and BPD severity grading [13]. The two-factor ANOVA analysis showed a significant difference in pulmonary V/Q by type of respiratory treatment (*p* = 0.011) and no difference across BPD subgroups (*p* = 0.30). Further, the interaction effect between the type of respiratory treatment and BPD severity grading on V/Q matching was not significant (*p* = 0.91). Altogether, these results indicate that the association found between the type of respiratory treatment and pulmonary V/Q matching outcome did not depend on BPD severity grading.

**Table 1:** Population perinatal characteristics, first week of life respiratory management variables, and outcome variables at 37 weeks postmenstrual age for the group of infants treated with high-frequency oscillatory ventilation (HFOV) compared with infants treated using controlled mechanical ventilation/continuous positive airway pressure (CMV/CPAP).

			HFOV *n* = 16	CMV/CPAP *n* = 14	*p* value
Perinatal characteristics					
Prenatal steroids	Full course		11	10	0.73
	Incomplete course		3	1	
	Missing data		2	3	
Cesarean section			9	9	0.72
Preterm premature rupture of membranes (pPROM)			7	0	0.007
Sex (male/female)			11/5	11/3	1.0
Small for gestational age			1	3	0.31
CRIB II score			10.5 (8.0, 14.0)	9.5 (7.0, 13.0)	0.48
Gestational age (weeks)			26.8 (25.1, 27.6)	27.5 (25.1, 28.7)	0.29
Birth weight (g)			1001 (719, 1161)	826 (700, 1189)	0.81
Weight z-score^a^			−0.23 (−0.89,0.17)	−1.06 (−2.02, −0.73)	0.01
First week of life, respiratory variables					
Started on CPAP			4	11	0.009
Any MV during first 7 days			16	12	0.21
Age at surfactant if given (hours of life)			0.1(0.1, 2.7)	3.2 (0.7, 10.2)	0.06
Received surfactant			16	11	0.09
OI at 12 h (%)			5.4 (4.2, 8.7)	2.5(2.0, 3.4)	<0.0001
OI at day 7 (%)			3.3(1.7, 10.0)	2.0(0.9, 5.2)	0.17
Maximum MAP Day 1 (cm H2O)			14 (11.7, 16)	7.4 (5.0, 8.7)	<0.0001
Maximum MAP Day 7 (cmH2O)			8.0 (4.0, 12.5)	6.0 (2.7, 10.1)	0.25
Variation maximum MAP Day 1 to 7 (cmH2O)			−5.3 (−8.0, −2.1)	−1.6 (−3.0, +2.7)	0.008
Maximum FiO2 day 1 (%)			71.5 (36.0, 100.0)	47.0 (35.8, 62.5)	0.26
Maximum FiO2 day 7 (%)			32.5 (25.5, 65.0)	31.0 (26.0, 36.2)	0.37
Days with MV first week			7 (4, 7)	4 (2.5, 7)	0.15
PaCO2 mean of daily high values day 1–7 (mmHg)			63.0 (60.8, 67.5)	56.2 (52.5, 63.0)	0.03
PaCO2 mean of daily lower values day 1–7 (mmHg)			42.7 (39.7, 45.7)	41.2 (38.2, 45.7)	0.98
36 weeks postmenstrual age, outcome variables					
Portion of the lung with matched V/Q-ratios (%)			60.4 (55.5, 66.0)	45.8 (37.8, 53.1)	0.01
Need for respiratory support		CPAP	6	4	0.43
		Low flow nasal cannula	4	7	
		No respiratory support	6	3	

Values are median and interquartile range (IQR).

*V/Q* = lung ventilation/perfusion matching, *CRIB* clinical risk index for babies, *MAP* mean airway pressure, *pCO2* partial pressure of carbon dioxide, *MV* mechanical ventilation, *FiO2* fraction of inspired oxygen, *OI* oxygenation index.

^a^Calculated using the reference values from Niklasson and Albertsson-Wikland [34].

**Table 2:** Treatment and secondary outcomes at discharge from the neonatal intensive care unit.

		HFOV *n* = 16	CMV/CPAP *n* = 14	*p* value
Days with mechanical ventilation		18.9	53.3	0 .71
Days with CPAP		38.9	32.6	0.36
Days with supplemental O2		115.2	137.1	0.53
Systemic postnatal steroids		3	4	0.67
IVH ≥ grade 3		1	0	1.0
NEC		2	3	0.64
Late-onset sepsis		11	6	0.27
ROP gr. 3 or more		3	3	1.0
BPD severity grading^a^	None	6	3	0.43
	Mild	4	7	
	Moderate	6	4	
	Severe	0	0	

^a^According to ref. [13].

ROP = retinopathy of prematurity, IVH = intraventricular hemorrhage, NEC = necrotizing enterocolitis.

### Perinatal presentation

Table 1 shows that the use of prenatal steroids, the proportions of cesarean sections, sex, gestational age, birth weight, CRIB II score and SGA were not statistically significantly different between infants in both treatment groups. Table 1 shows that the proportion of pPROM and the weight z-score were significantly higher for infants in the HFOV group as compared to the CMV/CPAP (*p* = 0.007 and *p* = 0.01, respectively). However, z-score showed a very week linear correlation with V/Q outcome; coefficient (*β*) = 3.33%, 95% confidence interval (CI) = [−0.48%, 7.14%], *p* = 0.08. Likewise, pPROM showed no correlation with V/Q outcome, *p* = 0.33 (Mann-Whitney). Further, as they were both added to a multivariate linear model, the relationship between V/Q outcome and type of respiratory treatment remained statistically significant, adjusted *β* = 12.42%, CI = [0.03%, 24.80%], *p* = 0.049, compared to the unadjusted model *β* = 12.98%, CI = [3.69%, 22.28%], *p* = 0.008.

### Respiratory variables associated with treatment the first week of life

Table 1 shows that significantly more infants initially starting on nasal CPAP were rescued with CMV as compared to HFOV (*p* = 0.009). Infants in the HFOV treatment group had a significantly higher oxygenation index (OI) at 12 h of age, higher maximal mean airway pressure (MAP) on day one and higher PaCO2 mean of daily high values on days 1–7, as compared to the CMV/CPAP group (Table 1). However, on day 7 no differences were found for OI and MAP between groups.

## Discussion

For infants in need of mechanical ventilation and who developed BPD, a high lung volume respiratory management strategy using HFOV and timely weaning over the ensuing 7 days, resulted in a statistically significantly better pulmonary V/Q matching at near-term age. In our analysis, the HFOV treatment group also had statistically significantly higher PaCO_2_ levels during the first week of life. These results can indicate that mechanical ventilation need the use of HFOV compared to CMV during the first week of life may be associated with less V/Q matching abnormalities at near-term age. To our knowledge, this observation has not been previously described.

At the time of recruitment, the patients received their BPD diagnosis and severity grading using the 2001 NIH workshop recommendations [12]. Interestingly, when we regrouped the patients according to the most recent definition [13], we find that the severity proportions changed. This cohort now consists of nine patients with no BPD (former mild), 21 patients with grade 1 or 2 BPD (former moderate or severe), and no grade 3 BPD (invasive mechanical ventilation). If BPD grading is not taken as a variable (Fig. 3), the group of patients treated with HFOV showed statistically significantly better pulmonary V/Q matching than patients treated with CMV/CPAP (*p* = 0.01). This significance was lost when stratifying the treatment groups according to their new BPD grading, most likely due to the small number of patients in each BPD subgroup (Fig. 4). Even if causality cannot be confirmed from our findings in this small group of infants, this associations indicate a need for further study on the effect of different respiratory management strategies on pulmonary V/Q matching in preterm infants.

Mechanical ventilation is a strong predictor for the development of BPD in preterm infants [4]. During MV excessively high tidal volumes and/or end-inspiratory lung volume are the main determinants of ventilator-induced lung injury [7]. Also, failure to initially open the lung homogeneously can result in atelectasis, focal overdistention, and cell damage [15]. In animal studies comparing HFOV with conventional ventilation, ventilation with HFOV used higher MAP initially but enabled a more rapid decrease in MAP and resulted in a lower oxygen requirement, less lung injury, less air leak, and a more uniform saccular expansion [16, 17].

In general, the aim of invasive mechanical respiratory support in extremely preterm infants is to limit the risk of lung overdistension and to control tidal volumes to avoid volutrauma. This can be achieved by using volume control during CMV or using high-frequency oscillatory ventilation (HFOV) [18]. During CMV, the benefits of volume guarantee compared with pressure-limited ventilation included shorter time on ventilation, lower occurrence of BPD, mortality, and IVH in preterm infants [19]. The optimal ventilation method for extremely preterm infants is not conclusive, although comparisons between HFOV employing a high lung volume strategy and different types of CMV have shown small improvements in the incidence of BPD, favoring HFOV [20]. Two recent reviews describe the pros and cons of HFOV treatment and the use of a high lung volume strategy [18, 21]

Studies have shown that hypocapnia compared to normocapnia increases the risk for BPD, even in infants with relatively good pulmonary function [22]. Permissive hypercapnia has been suggested to decrease ventilation-induced lung injury [23]. It is commonly used but the optimal PaCO_2_ level is still unknown [24]. However, high, and fluctuating values of PaCO_2,_ are also risk factors for IVH/death, BPD/death, and neurodevelopment impairment/death in extremely preterm infants [25]. The levels of hypercapnia we observed may represent less aggressive ventilatory management with smaller tidal volumes minimizing the risk for volutrauma.

The incidence of CPAP failure is high in randomized controlled studies comparing invasive and non-invasive respiratory support [3]. It is often characterized by increasing oxygen demand most likely related to poor functional residual capacity (FRC) control, increasing the risk for lung injury, especially in the most immature infants. In our cohort, all but two patients who started on CPAP failed. One interpretation, based on the significantly better V/Q results for patients treated with HFOV would be that after CPAP failure, HFOV as a rescue mode is preferable to recover lung volume. Similarly, a HFOV lung recruitment maneuver used shortly before surfactant installation in extremely preterm infants failing CPAP resulted in reduced MV need most probably due to an improved surfactant response after the recruitment procedure [26].

### Limitations of the study

In healthy adults, children, and healthy newborns, the expected proportion of the lung with V/Q matching within normal range [0.6–1.4] is ~85%, but reference values for healthy preterm infants are lacking [27, 28]. Lower values for the patients classified as no BPD were found in this cohort, around 60% in the no BPD group (Fig. 4), but in the same range as those described by others [10, 11, 29]. Whether these results are “normal” is debatable as some degree of V/Q abnormalities are still seen across the whole severity spectrum of BPD, including the current no BPD grade which was the former mild BPD grade. Being born preterm has been correlated with overall worse lung function in otherwise healthy preterm-born infants [30]. Also, longitudinal data on lung function (mechanics and gas exchange) up to 12 years of age in infants and children born before 32 weeks gestation with or without BPD found the largest lung function trajectory impairment in BPD survivors [31]. The long-term positive effects of a lung protective ventilation strategy may not appear early. To demonstrate this a follow-up in children who participated in the United Kingdom Oscillation trial, using a high volume HFOV strategy or CMV, initially showed no difference in the incidence of BPD at 36 weeks corrected age or in lung function at 1 year of age [32]. However, at 11–14 years of age follow-up there was a statistically significant better outcome in all parameters of small airway function favoring the HFOV treatment group [33]. In adult’s SPECT has shown a better ability to find diffusion abnormalities despite normal traditional lung function tests [9]. We speculate that using a ventilation strategy which minimizes the extent of early V/Q abnormalities may improve the odds for better functional lung volume with advancing age when evaluated with SPECT.

Because of the observational nature of this study, a major limitation is that the infants in the two treatment groups could not be properly matched with regards to baseline characteristics. However, as Table 1 shows, all recorded perinatal variables except pPROM and weight z-score at birth did not differ between treatment groups and the multivariate analysis showed that the association between V/Q and respiratory treatment strategy remain after adjusting for these factors. Still, due to the design of this study and the small number of patients, confounding cannot be completely ruled out. We therefore suggest that special care should be taken in further randomized studies regarding the possible influence of these perinatal variables. Table 1 also showed that the factors related with the choice of respiratory treatment including OI, PaCO2 at 12 h and maximum MAP on day 1 were also significantly higher for the HFOV group as compared to the CMV/CPAP. This invites an interpretation that the infants in the HFOV group had more severe lung disease at the beginning and should also be considered confounders in the multivariate analysis. However, the rationale behind not including these variables in the multivariate analysis was as follows:

(i) The OI is the product of FiO2, airway pressure, and PaO2. When using HFOV, initial OI will almost always be higher for any given PaO2 because the distending pressures (CDP) used are static and higher than the calculated mean airway pressure in CMV. Thus, the OI value for infants in the HFOV group who were started on invasive HFOV dominates the value and does not necessarily define worse lung disease. This is also illustrated by the significantly higher MAP at 12 h.
(ii) The degree of permissive hypercapnia is chosen by the operator and is more easily controlled in HFOV. The aim is to use an amplitude smaller than dead space and allow permissive hypercapnia to minimize the risk for lung damage from volutrauma.

A unique strength in our study is the use of SPECT which enables the quantification of V/Q abnormalities at a regional pulmonary level. Our study can then be used as a rationale for future studies investigating the effect of different respiratory care strategies on V/Q as well as the longitudinal development of V/Q in BPD survivors.

## Conclusions

In infants who needed mechanical ventilation the first week of life and later developed BPD we observed a positive association between treatment with a HFOV high lung volume strategy and better pulmonary V/Q matching at near term age. The nature of these findings based on an observational study should then be regarded as a rationale for future randomized prospective studies.

### Summary

#### What is already known on this topic?

- The regional distribution of the pulmonary V/Q matching defects can be studied noninvasively with SPECT.
- Pulmonary V/Q matching defects are found in infants and children with BPD.
- Predictors for the development of BPD include mechanical ventilation.

#### What this study adds

- Treatment with a HFOV high lung volume strategy was positively associated with better pulmonary V/Q matching at near term age in premature patients who needed mechanical ventilation and developed BPD.

## Data Availability

The datasets generated during and/or analyzed during the current study are not publicly available but are available from the corresponding author on reasonable request.
